# Power Gain from Energy Harvesting Sources at High MPPT Sampling Rates

**DOI:** 10.3390/s23094388

**Published:** 2023-04-29

**Authors:** Manel Gasulla, Matias Carandell

**Affiliations:** Electronic Engineering Department, Universitat Politècnica de Catalunya, C/Jordi Girona 31, 08034 Barcelona, Spain; matias.carandell@upc.edu

**Keywords:** energy harvesting, maximum power point tracking (MPPT), power gain, power management unit, wireless sensor

## Abstract

Energy harvesting (EH) sources require the tracking of their maximum power point (MPP) to ensure that maximum energy is captured. This tracking process, performed by an MPP tracker (MPPT), is performed by periodically measuring the EH transducer’s output at a given sampling rate. The harvested power as a function of the sampling parameters has been analyzed in a few works, but the power gain achieved with respect to the case of a much slower sampling rate than the EH source’s frequency has not been assessed so far. In this work, simple expressions are obtained that predict this gain assuming a Thévenin equivalent for the EH transducer. It is shown that the power gain depends on the relationship between the square of AC to DC open circuit voltage of the EH transducer. On the other hand, it is proven that harvested power increases, using a suitable constant signal for the MPP voltage instead of tracking the MPP at a low sampling rate. Experimental results confirmed the theoretical predictions. First, a function generator with a series resistor of 1 kΩ was used, emulating a generic Thévenin equivalent EH. Three waveform types were used (sinus, square, and triangular) with a DC voltage of 2.5 V and AC rms voltage of 0.83 V. A commercial MPPT with a fixed sampling rate of 3 Hz was used and the frequency of the waveforms was changed from 50 mHz to 50 Hz, thus effectively emulating different sampling rates. Experimental power gains of 11.1%, 20.7%, and 7.43% were, respectively, achieved for the sinus, square, and triangular waves, mainly agreeing with the theoretical predicted ones. Then, experimental tests were carried out with a wave energy converter (WEC) embedded into a drifter and attached to a linear shaker, with a sinus excitation frequency of 2 Hz and peak-to-peak amplitude of 0.4 g, in order to emulate the drifter’s movement under a sea environment. The WEC provided a sinus-like waveform. In this case, another commercial MPPT with a sampling period of 16 s was used for generating a slow sampling rate, whereas a custom MPPT with a sampling rate of 60 Hz was used for generating a high sampling rate. A power gain around 20% was achieved in this case, also agreeing with the predicted gain.

## 1. Introduction

Wireless sensors are key components of the Internet of Things [1]. They are commonly powered by primary batteries although energy harvesting (EH) has proven to be a reasonable alternative. Batteries have a simple design but their energy is limited [2]. Contrariwise, energy harvesters provide unlimited energy, reducing the maintenance and associated costs of battery-powered wireless sensors at the expense of a more complex design. Mainly, a power management unit (PMU) is required to adapt the random nature of the EH transducer to a constant and clean output and to control the mismatch of energy between the transducer and the wireless sensor.

One key block of PMUs is the maximum power point tracking (MPPT) module [3], which aims to extract maximum power from the EH transducer. It consists of a power converter and a tracking algorithm. The power converter mainly consists of a DC–DC converter, but, for AC signals, e.g., those coming from mechanical or radiofrequency transducers, a previous AC–DC rectifier is required. The tracking algorithm provides the reference required to fix the output voltage of the EH transducer (or that of the rectifier) at its maximum power point (MPP).

Two widespread MPPT algorithms are the fractional open circuit voltage (FOCV) and the perturb and observe (P&O). The FOCV is based on the ratio (*k*) between the MPP voltage (*v*_MPP_) and the open circuit voltage (OCV) of the EH transducer, e.g., 0.5 for thermoelectrical generators and between 0.7 and 0.8 for solar cells. FOCV methods are usually implemented by periodically opening the EH transducer for a short sampling time (*t*_SAMP_), measuring its OCV, and fixing the new *v*_MPP_. This technique is simple but the true MPP is not ensured. Contrariwise, the P&O performs the periodic measurement of the EH transducer’s output power. The true MPP is achieved at the cost of increased complexity. Anyhow, a periodic measurement at a given sampling period (*T*_s_) is required in these and other methods. Its inverse (*f*_s_ = 1/*T*_s_) is defined as the MPPT sampling rate.

The sampling rate must be high enough to follow the fluctuation of the EH source to continuously place the EH transducer at its MPP. Light and thermal sources are usually slowly varying. On the other hand, mechanical sources, e.g., vibrations, can have relatively fast fluctuations. For example, refs. [4,5] present a wind energy harvester (WEH) and a wave energy converter (WEC), respectively, each with the OCV oscillating at around 1.8 Hz. WECs are commonly used to expand the autonomy of free-floating monitoring buoys (e.g., drifters) and many of them have been recently reported. Reference [6] describes an electromagnetic converter that captures energy from the relative motion between a drogue and a drifter, achieving tens of milliwatts of average power in a simulation test. Reference [7] presents an electromagnetic-based swing body that achieved power peaks of 0.13 W under real waves of 0.8 m height. A small-sized, pendulum-type, and electromagnetic-based WEC was reported in [8], harvesting energy from a 20 cm diameter drifter, achieving an average useful power of 0.2 mW. These studies show the potential of electromagnetic converters as a promising approach to generate energy from ocean waves, as long as the MPP of its oscillating output is tracked fast enough.

Most of the commercial MPPT-based integrated circuits (ICs) use techniques based on the simple FOCV method, with a *T*_s_ of several seconds, so not appropriate for these fast-varying EH sources. As an example, the BQ25504/5 (Texas Instruments) and the ADP5091/2 (Analog Devices) are two of the most widely used ICs. Their DC–DC converters are very efficient (>80%) and work in a wide range of powers (from µW to mW) from low input voltages (<100 mV). However, *T*_S_ is fixed to 16 s, which is too slow for fast-varying EH sources. Conversely, there are several academic proposals where the sampling rate is high enough. References [9,10] use the FOCV method for PV sources, reporting *T*_s_ values of 100 ms and 3.33 ms, respectively. Reference [11] examines a vibrational EH source that employs a piezoelectric device, with the PMU refreshing the FOCV–MPPT after the PZT voltage rectification step with *T*_s_ = 1 s. In [12], a boost converter is used to harvest energy from PV cells using the FOCV method with very low input voltages and *T*_S_ = 150 ms. Reference [13] presents a FOCV technique with an adaptative sampling period that can be reduced down to 4 ms. Other fast-tracking MPPT methods have also been reported, as presented in [14], where the P&O method has been used for PV sources to feed wireless sensor nodes.

Nevertheless, few works have analyzed the harvested power in function of the sampling parameters. In [15], the sampling parameters are optimized for the FOCV and the P&O methods to maximize the power harvested from resonant piezoelectric vibration harvesters (RPVH) after an AC–DC bridge rectification step. Expressions are provided and experimentally validated for a 153.6 Hz sinusoidal acceleration whose amplitude is modulated by a 50 s period saw-tooth waveform with acceleration ranging from 0.75 g to 1.25 g. For the FOCV method, an optimum *T*_s_ of 16.7 s with a *t*_SAMP_ of 0.3 s is the result. An approximate analytical expression is provided in [16] for the harvested power with the FOCV method, considering a sinusoidal waveform for the EH transducer. It is shown that for negligible sampling times, the larger the sampling rate, the larger the harvested power, and that 99% of the maximum power can be obtained with a sampling rate just 15 times that of the EH transducer’s frequency. On the other hand, the same as in [15], a trade-off exists for non-negligible sampling times resulting in an optimum sampling rate. Experimental tests made with a WEC under simulated sea condition were also performed.

Nonetheless, to the best of our knowledge, no work in the literature theoretically estimates the power gain achieved by sampling at high rates with respect to the case of low sampling rates. For this, it is necessary to tackle both the favorable case when *f*_s_ is much higher than the EH source frequency (*f*_o_), i.e., *f*_s_ >> *f*_o_ (high sampling rate), as well as the unfavorable case when *f*_s_ << *f*_o_ (low sampling rate) and thus the harvested power decreases. This paper provides in both cases simple expressions for the harvested power of time-varying EH sources, assuming they can be modelled as a Thévenin equivalent, and the corresponding power gain. On the other hand, it is demonstrated that more power can be harvested by setting a suitable constant value for *v*_MPP_ instead of tracking the MPP at a low sampling rate. Experimental results corroborate the analytical findings. First, a function generator (FG) was used to emulate a generic Thévenin equivalent EH transducer. Then, an actual WEC attached to a linear shaker provided more realistic results.

## 2. Theoretical Analysis

The proposed analysis derives the harvested power from EH transducers, both for high and low sampling rates, with respect to the frequency of the EH transducer signal (*f*_s_/*f*_o_), and the corresponding power gain that can be achieved. The analysis is circumscribed to EH transducers that can be modelled as a Thévenin (or Norton) equivalent circuit. This is the case, for example, of thermoelectric transducers and DC electrical generators as in [17] and [8], respectively, but also of radiofrequency [18] and resonant piezoelectric vibration energy harvesters [19] when including the AC–DC rectifier.

**Figure 1:**
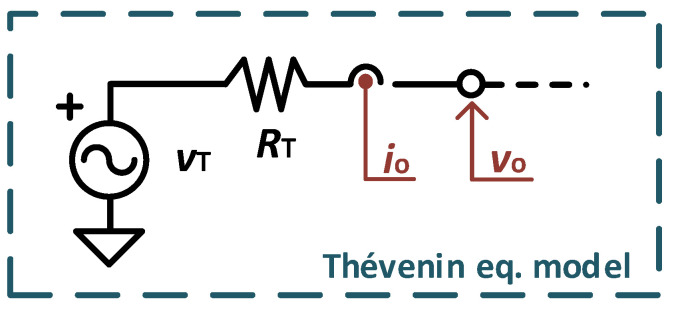
Thévenin equivalent circuit and parameters.

Figure 1 shows the Thévenin equivalent circuit of the energy transducer, where *v*_T_ is the Thévenin voltage (OCV), *R*_T_ is the Thévenin resistance, and *v*_o_ and *i*_o_ are the output voltage and current, respectively. It is well known that the output power, *p*_o_ = *v*_o_×*i*_o_, can achieve a maximum value whenever the equivalent resistance connected to the output terminals is equal to *R*_T_, or equivalently when *v*_o_ = *v*_MPP_ = *v*_T_/2 [20]. In this case, *p*_o_ is given by

(1) $$p_{MPP}=v_{T}^{2}/4R_{T}$$

EH source variations will translate to variations in *v*_T_. So, *v*_T_ can be expressed as

(2) $$v_{T}\left(t\right)=V_{dc}+v_{ac}\left(t\right)$$

where *V*_dc_ and *v*_ac_ are the DC (average) and AC (time-varying) components of *v*_T_. Substituting Equation (2) into Equation (1) and performing the time average, we obtain the average of *p*_MPP_

(3) $$P_{MPPH}=\bar{p_{MPP}\left(t\right)}=\frac{V_{dc}^{2}+V_{rms}^{2}}{4R_{T}}=P_{dc}\left(1+\alpha^{2}\right)$$

where *V*_rms_ is the rms voltage of *v*_ac_, *P*_dc_ = $V_{dc}^{2}/4R_{T}$ and $\alpha =V_{rms}/V_{dc}$. *V*_dc_ can take positive and negative values whereas V_rms_ only takes positive values. So, *α* can take any positive or negative value, becoming infinite for *V*_dc_ = 0. For the particular case in which *v*_ac_ is a sinus, triangular, or square signal, *V*_rms_ is *V*_p_/√2, *V*_p_/√3, or *V*_p_, respectively, where *V*_p_ is the peak voltage of *v*_ac_.

**Figure 2:**
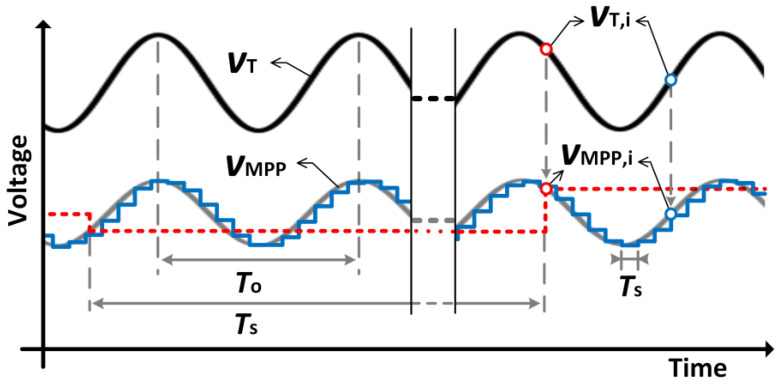
Sinusoidal-shape, time-varying *v*_T_ signal and the corresponding *v*_MPP_ (=*v*_T_/2) together with the resulting *v*_o_ for two sampling rates: *T*_s_ << *T*_o_ (blue solid line) and *T*_s_ >> *T*_o_ (red dashed line).

To fix the MPP, an MPPT algorithm must be used, e.g., FOCV or P&O, that periodically samples *v*_T_ (every *T*_s_). Figure 2 shows an illustrative example where *v*_T_ is represented as a sinus with positive offset and period *T*_o_ (=1/*f*_o_). Additionally, the corresponding *v*_MPP_ (=*v*_T_/2), together with the resulting *v*_o_ for high (*T*_s_ << *T*_o_) and low sampling rates (*T*_s_ >> *T*_o_), is also shown. As can be seen in both cases, *v*_o_ is fixed to *v*_MPP,*i*_ = *v*_T,*i*_/2 each *T*_s_ (the subindex *i* indicates the sampling number), whereas *v*_T_ keeps varying.

The result in Equation (3) is achieved for the case of high sampling rates. In [16], it is shown that 99% of the maximum power is achieved for *f*_s_ = 15*f*_o_. For any waveform type, Equation (3) is achieved whenever *f*_s_ >> *f*_omax_, with *f*_omax_ being the maximum frequency component of *v*_ac_. On the other hand, for low sampling rates and assuming for the sake of simplicity that *T*_s_ is an integer multiple of *T*_o_, the average of *p*_MPP_ within *T*_s_ will be

(4) $$\bar{p_{MPP,i}\left(t\right)}=\frac{1}{T_{s}}\int_{T_{s}}^{}v_{MPP,i}\left(v_{T}\left(t\right)-v_{MPP,i}\right)/R_{T}=v_{MPP,i}\left(V_{dc}-v_{MPP,i}\right)/R_{T}$$

This expression is valid for any *v*_T_ periodic signal. The value of Equation (4) depends on the sampled value *v*_MPP,*i*_. Zero values are achieved for *v*_MPP,*i*_ equal to 0 or *V*_dc_ (*v*_T,i_ equal to 0 or 2*V*_dc_) and even negative values surpassing these limits, whenever the excursion range of *v*_T_ allows it. Differentiating Equation (4) with respect to *v*_MPP,*i*_ and equating it to zero, we obtain *v*_MPP,*i*_ = *V*_dc_/2 (*v*_T,i_ = *V*_dc_), for which the power is maximum and equal to

(5) $$\bar{p_{MPP,i}\left(t\right)}=P_{dc}$$

Yet, this maximum value is lower than Equation (3) since the sampled value is held constant and not is following the pace of *v*_T_. 

In a realistic scenario, *T*_s_ will not be a multiple of *T*_o_, and *v*_MPP,*i*_ will take values within the full range of *v*_T_/2. In the long term, all the instants within a period *T*_o_, where the measurements every *T*_s_ are performed, are equiprobable. Thus, the overall average of *p*_MPP_ can be obtained performing the time average of Equation (4), resulting in

(6) $$\bar{p_{MPP}\left(t\right)}=\overset{¯}{\overset{¯}{p_{MPP,i}\left(t\right)}}=\frac{\bar{V_{T}\left(V_{dc}-\frac{V_{T}}{2}\right)}}{2R_{T}}=P_{dc}\left(1-\alpha^{2}\right)$$

where *v*_MPP,*i*_ has been substituted by *v*_T_/2, and *v*_T_ is given by (2). The result of (6) can be extrapolated to any waveform type as long as *f*_s_ << *f*_omin_, where *f*_omin_ is the minimum frequency component of *v*_ac_. 

As can be seen, Equation (3) is higher than Equation (6). This means that using low sampling rates decreases the harvested power. In fact, for α > 1, Equation (6) becomes negative, which means that the EH transducer, on average, would drain the power instead of producing it. The value of Equation (5) is also higher than Equation (6). So, whenever the sampling rate cannot be conveniently increased, a better strategy is to fix *v*_MPP_ to *V*_dc_/2 to achieve Equation (5). Moreover, Equation (4) is also higher than Equation (6) whenever

(7) $$\left(V_{dc}-V_{rms}\right)/2<v_{MPP,i}<\left(V_{dc}+V_{rms}\right)/2$$

However, the knowledge of *V*_dc_ (and *V*_rms_) requires a previous characterization of the EH source and transducer. To assess the benefit of increasing the sampling rate, a normalized power gain factor (*G*_p_) is defined as the difference between Equations (3) and (6) divided by *P*_dc_

(8) $$G_{p}=\frac{P_{MPPH}-P_{MPPL}}{P_{dc}}=2\alpha^{2}$$

The value of *P*_dc_ can also be obtained as the average of *P*_MPPH_ and *P*_MPPL_. Whenever *v*_T_ is a purely DC signal, i.e., *V*_rms_ and thus *α* are zero, Equations (3) and (6) are equal to *P*_dc_ and *G*_P_ = 0, so that no power gain is achieved by operating at high sampling rates. When |*α*| is increasing, *G*_p_ increases too, and thus using high sampling rates makes sense. The larger the |*α*|, the larger the power gain. An infinite value of |*α*| and *G*_p_ is achieved for *V*_dc_ = 0, i.e., for *v*_T_ with no DC value because *P*_dc_ becomes zero.

## 3. Materials and Methods

Two setups and tests were used and performed to validate the analytical findings. First, an FG was used to emulate a generic Thévenin equivalent EH transducer. Then, an actual WEC attached to a linear shaker emulated a drifter’s movement under a sea environment. 

### 3.1. Test with a Function Generator

**Figure 3:**
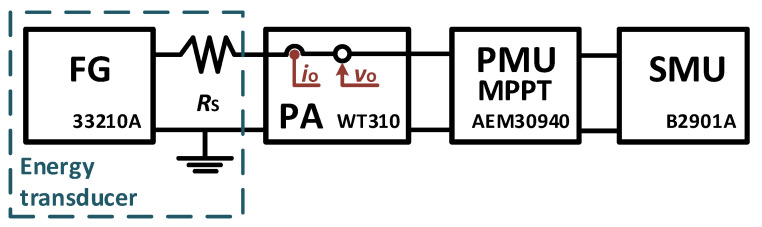
Experimental setup for the test with the function generator.

Figure 3 shows the experimental setup of the first test used to prove the formulation of Section 2. The EH transducer was emulated with a function generator, FG (33210A, Agilent; output impedance of 50 Ω) in series with a resistor of 1 kΩ (*R*_S_), and thus *R*_T_ = 1,05 kΩ. As for the MPPT, the evaluation board of the AEM30940 PMU chip (e-peas) was used. It implements the FOCV technique with *T*_s_ = 0.33 s (*f*_s_ = 3 Hz) and *t*_SAMP_ = 5.12 ms. The value of *k* was set to 0.5. A power analyzer, PA (WT310, Yokogawa), was placed between the FG and the MPPT to measure the input power. The PA was programmed with an integration time of 100 s, accounting for 300 samples of the MPPT (300*T*_s_). The PMU output (BATT pin) was connected to a source measure unit, SMU (B2901A, Agilent), fixed at 3.9 V.

The MPPT only accepts positive values of *v*_o_ and *i*_o_, and therefore of *p*_o_. Thus, *v*_T_ was set positive and its minimum value (*v*_Tmin_) was set higher than the maximum value of *v*_MPP,*i*_ (*v*_MPPmax_) to keep *i*_o_ positive. Hence, the following inequality must be satisfied for periodic signals:

(9) $$v_{Tmin}=V_{dc}-V_{p}>v_{MPPmax}=\frac{V_{dc}+V_{p}}{2}\Rightarrow V_{p}<V_{dc}/3$$

Accordingly, the FG was programed with *V*_dc_ = 2.5 V and *V*_p_ = 0.83 V (*α* = 0.236). Three waveforms types were used: sinus, square, and triangular. Thus, we have *P*_dc_ = 1.488 mW and for the sine/square/triangular waveforms: *P*_MPPH_ = 1.571/1.653/1.543 mW from Equation (3), *P*_MPPL_ = 1.405/1.323/1.433 mW from Equation (6), and *G*_p_ = 11.1/22.2/7.41% from Equation (8). Frequency *f*_o_ was swept over 3 decades from 50 mHz (*T*_o_ = 20 s) to 50 Hz (*T*_o_ = 20 ms) in a sequence 1-2-5-10. The resulting *f*_s_/*f*_o_ ranged from 0.06 (low sampling rate) to 60 (high sampling rate). 

### 3.2. Test with a WEC

**Figure 4:**
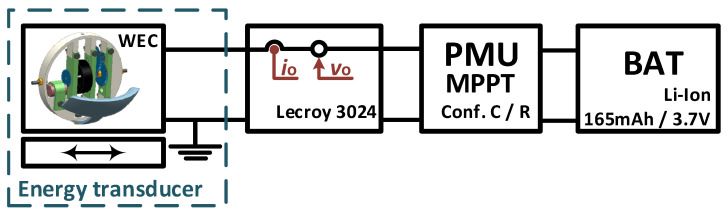
Scheme of the experimental setup for the WEC test.

**Figure 5:**
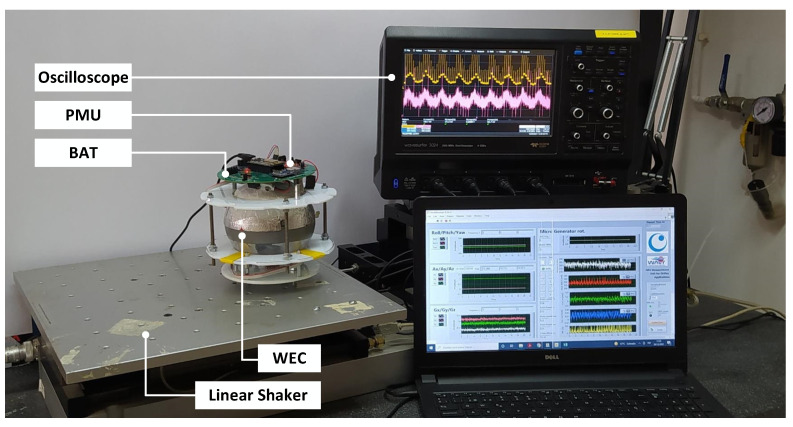
Picture of the experimental setup for the WEC test.

In [16], experimental tests were carried out with a WEC embedded into a drifter and attached to a linear shaker (APS 129), with an excitation frequency of 2 Hz (*f*_o_), in order to emulate the drifter’s movement under a sea environment (Section V.C of [16]). A block schematic and a picture of the experimental setup are shown in Figure 4 and Figure 5, respectively. The electrical model for the WEC matches that of Figure 1. The WEC was attached to the shaker’s moving platform with the device’s pendulum aligned to the movement axis. The shaker’s acceleration was set with a sinus wave of frequency 2 Hz and peak-to-peak amplitude of 0.4 g, similar to that reported in [5] from a drifter under sea-wave excitation. The WEC’s output was connected to the PMU. Two MPPT systems were used, both using a FOCV method. First, the commercial ADP5092 IC with a low sampling rate (config. R: *f*_s_ = 1/16 Hz = *f*_o_/32) was used. Second, a custom PMU using the ADP5092 IC with additional low-power sampling circuitry to drastically increase the sampling rate with respect to config. R. (config. C: *f*_s_ = 60 Hz = 30*f*_o_) was used. A Li-ion rechargable battery of 165 mAh and 3.7 V was placed as a load at the PMU’s output. An oscilloscope (Lecroy Wavesurfer 3024) was used to measure both *v*_o_ and *i_o_* (this last one also using a shunt resistor and a current sense amplier as described in [16]). From these parameters, input power to the PMU can be estimated. The data obtained in [16] were used here with further processing to validate the equations presented here for high and slow MPPT sampling rates.

## 4. Results and Discussions

### 4.1. Test with a Function Generator

**Figure 6:**
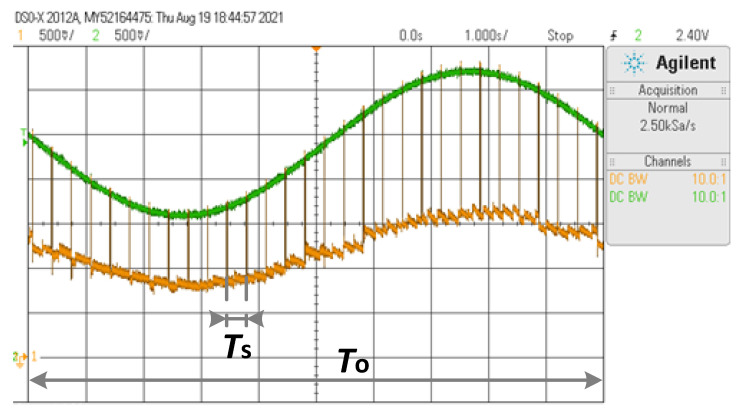
Oscilloscope screen capture for the sine waveform when *f*_o_ = 0.1 Hz. CH1: *v*_o_—500 mV/div, CH2: *v*_T_—500 mV/div, and time base 1 s/div.

**Figure 7:**
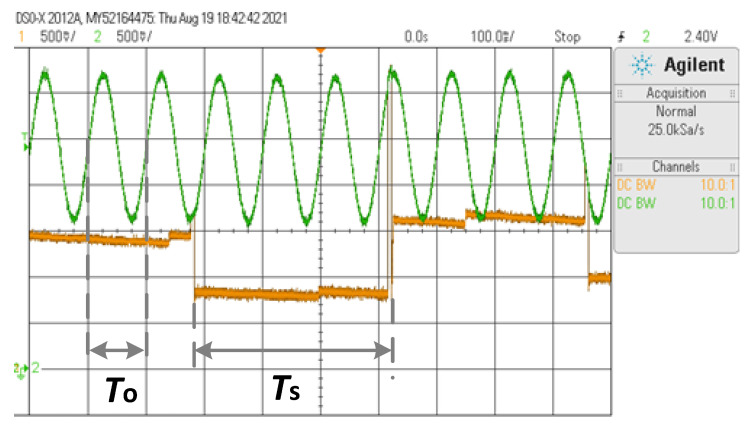
Oscilloscope screen capture for the sine waveform when *f*_o_ = 10 Hz. CH1: *v*_o_—500 mV/div, CH2: *v*_T_—500 mV/div, and time base 100 ms/div.

Figure 6 and Figure 7 show, for the sine waveform, oscilloscope screen captures of *v*_o_ (in orange) and at the output of the FG (in green) for *f*_o_ = 0.1 Hz and *f*_o_ = 10 Hz, respectively. The output of the FG nearly provides *v*_T_. In both cases, the sampling process happens every 0.33 s approximately, as previewed, where *v*_o_ instantly rises to *v*_T_ and then settles to the updated value (*v*_T_/2). For *f*_o_ = 0.1 Hz (*f*_s_/*f*_o_ = 30), *v*_o_ nearly follows *v*_T_/2, whereas for *f*_o_ = 10 Hz (*f*_s_/*f*_o_ = 0.3), *v*_o_ cannot keep the pace of *v*_T_.

Table 1 shows *f*_o_, *f*_s_/*f*_o_ and the experimental results of *p*_o_ for the three waveform types. As can be seen, *p*_o_ approaches the predicted values both for high (*f*_s_/*f*_o_ >> 1) and low sampling rates. Experimental values are slightly lower, which can be justified by the non-negligible value of *t*_SAMP_ with respect to *T*_s_ (1.55%) during which no energy is harvested [16]. In between, a minimum is found around *f*_s_/*f*_o_ = 1.5. The experimental values of *G*_P_ are obtained from Equation (8) using the power values of the first row as *P*_MPPL_ and those of the last row as *P*_MPPH_. *P*_dc_ was calculated from the average of these two values. The resulting values are *G*_p_ = 11.1/20.7/7.43%, thus mainly agreeing with the theoretical ones. Larger values of *G*_p_ could be achieved by increasing *V*_p_ and thus *α*. However, as stated in Section 3.1, this was not implemented by the limitations of the MPPT chip. 

### 4.2. Test with a WEC

**Figure 8:**
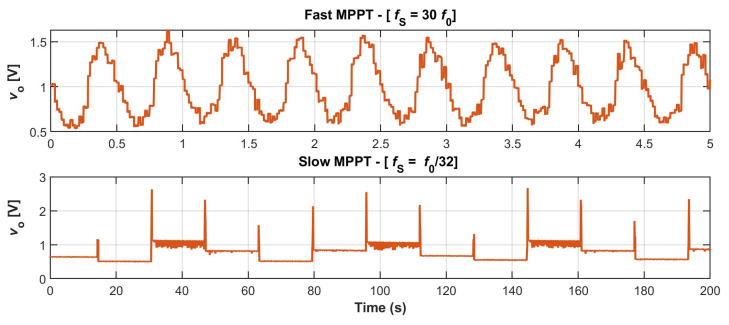
Measured *v*_o_ for the WEC test. (**Top**): fast MPPT (config. C). (**Bottom**): slow MPPT (config. R).

Figure 8 shows the measured *v*_o_ for the WEC test using configurations C (fast MPPT) and R (slow MPPT). For the fast MPPT, an acquisition window of 5 s was used, whereas for the slow MPPT, it was set to 200 s. For the slow MPPT, *v*_o_ increases to *v*_T_ every *T*_s_ = 16 s during *t*_SAMP_ = 256 ms. In the fast MPPT, the voltage is nearly sinusoidal and corresponds to *V*_T_/2. In this case, *v*_o_ does not rise to *V*_T_ on each sample, as usual, which is a particularity of config. C [16]. From these last data, we can process the values of *V*_dc_ and *V*_rms_, which are 2.006 V and 0.616 V, respectively. Thus, from Equation (3), *P*_MPPH_ = 8.67 mW, and from Equation (6), *P*_MPPL_ = 7.17 mW. These values nearly match those experimentally measured in [16] with the fast and slow MPPT systems, 8.66 mW and 6.93 mW, respectively. The corresponding theoretical and experimental values of *G*_p_ are 18.9% and 22.2%, respectively. So, a good match is also achieved with an actual EH transducer.

## 5. Conclusions

This work shows that by increasing the sampling rate of MPPTs, more power can be extracted from the energy transducers. In particular, the sampling rate should be quite a lot higher, e.g., at least 15–30 times, than the frequency of the EH source. Contrariwise, sampling at low rates, lower than the frequency of the EH source, is detrimental. A normalized power gain factor has been defined resulting in a simple analytical expression, which depends on the relationship of the square of the AC to DC voltage of the EH source, which is assumed as an equivalent Thévenin circuit. Experimental results have confirmed the theoretical predictions. A generic Thévenin equivalent EH source has been emulated using an FG programmed with sinusoidal, square, and triangular waveform types. The DC voltage was set to 2.5 V and the AC RMS voltage to 0.83 V in all cases. A commercial MPPT system with a sampling rate of 3 Hz has been used to measure the power gains achieved by varying the frequency of the FG across three decades, from 50 mHz to 50 Hz. The power gains obtained have been 11.1%, 20.7%, and 7.43% for sinusoidal, square, and triangular waves, respectively, which are in agreement with theoretical predictions. Additionally, experimental tests have been conducted with a WEC embedded into a drifter and attached to a linear shaker, mimicking the drifter’s movement under a sea environment, with an excitation frequency of 2 Hz and a peak-to-peak amplitude of 0.4 g. The WEC provides a sinus-like wave. A commercial MPPT with a sampling period of 16 s has been used to fix a low sampling rate, whereas a custom MPPT with a sampling rate of 60 Hz generates a high sampling rate. This results in a power gain of around 20%. Therefore, the expressions presented in this study provide a useful tool for predicting the power gain that can be achieved by choosing an appropriate sampling rate for the MPPT system. This can help to optimize the performances of MPPT systems in future studies.

## Figures and Tables

**Table 1:** Experimental values for the test with a function generator.

*F*_o_ (Hz)	*f*_s_/*f*_o_	Sinus *p*_o_ (mW)	Square *p*_o_ (mW)	Triangle *p*_o_ (mW)
50	0.06	1.385	1.314	1.412
20	0.15	1.386	1.310	1.412
10	0.3	1.395	1.327	1.418
5	0.6	1.374	1.294	1.406
2	1.5	1.357	1.267	1.395
1	3	1.454	1.417	1.457
0.5	6	1.520	1.527	1.501
0.2	15	1.543	1.589	1.517
0.1	30	1.546	1.609	1.520
0.05	60	1.547	1.617	1.521

## Data Availability

Not applicable.
